# Objective measurement of nine gaze-directions using an eye-tracking device

**DOI:** 10.16910/jemr.13.6.4

**Published:** 2020-10-06

**Authors:** Yo Iwata, Tomoya Handa, Hitoshi Ishikawa

**Affiliations:** Department of Rehabilitation, Orthoptics and Visual Science Course, School of Allied Health Sciences, Kitasato University, Sagamihara, Japan; Department of Ophthalmology, School of Medicine, Kitasato University, Sagamihara, Japan; These authors contributed equally

**Keywords:** Eye movement, nine gaze-directions, eye tracking, objective measurement, gaze, individual differences

## Abstract

Purpose: To investigate the usefulness and efficacy of a novel eye-tracking device that can objectively measure nine gaze-directions.

Methods: We measured each of the nine gaze-directions subjectively, using a conventional Hess screen test, and objectively, using the nine gaze-direction measuring device, and de-termined the correlation, addition error, and proportional error. We obtained two consecu-tive measurements of the nine gaze-directions using the newly developed device in healthy young people with exophoria and investigated the reproducibility of the measurements. We further measured the nine gaze-directions using a Hess screen test and the newly developed device in three subjects with cover test-based strabismus and compared the results. Results: We observed that the objective measurements obtained with the newly developed gaze-direction measuring device had significant correlation and addition error compared to the conventional subjective method, and we found no proportional error. These measure-ments had good reproducibility.

Conclusion: The novel device can be used to observe delayed eye movement associated with limited eye movement in the affected eye, as well as the associated excessive movement of the healthy eye in patients with strabismus, similar to the Hess screen test. This is a useful device that can provide objective measurements of nine gaze-directions.

## Introduction

The Hess screen test is an extremely useful test for quantifying limited eye movement by measuring ocular deviations in each of the nine gaze-directions and for identifying paretic muscles; it is frequently implemented in clinical ophthalmology 1). The most important feature of the Hess screen test is that it can visually capture the patient’s eye movement in the nine gaze-directions. However, the conventional Hess screen test has various drawbacks. Firstly, a tester is required for each description of the results, thus results may contain an information bias. Furthermore, the Hess screen test cannot be used for measuring patients with suppression because the grid is displayed in one eye and the target is displayed in the other eye under the red–green dichoptic separation (2). Although the Hess screen test is mainly used in patients with strabismus, these patients often develop the suppression phenomenon, rendering the test unsuitable (3). Also, since this is a subjective test, patient response is essential.

We have previously developed an objective eye-position testing device for the central eye position, using gaze-detection technology (4). Gaze-detection technology has recently undergone remarkable development, and it is now possible to detect the gaze using a highly precise compact device (5, 6, 7). Therefore, in this study we have newly developed a device that can objectively measure the nine gaze-directions using gaze-detection technology and compare the findings obtained with this device to those from the Hess test, in patients with strabismus.

## Methods

The study conformed to the tenets of the Declaration of Helsinki and was approved by the Kitasato University Human Sciences Ethics Committee (C-18-009). All procedures were carried out in accordance with approved guidelines. Informed consent was obtained from all subjects after the nature and possible consequences of the study had been explained to them. The clinical study registration number is UMIN000033881 (UMIN - CTR), and the registration date is August 24, 2018.

**Figure 1.**
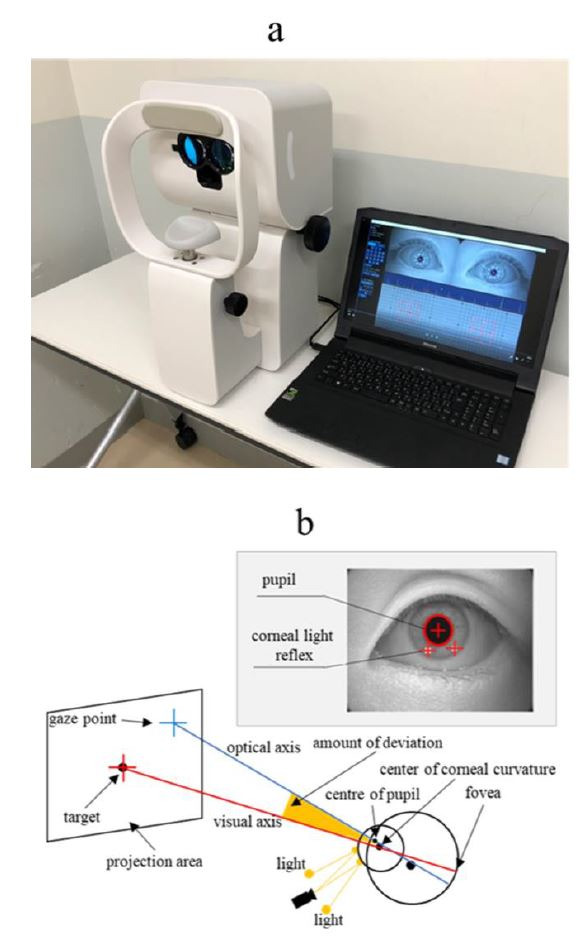
a: External view of the OMD; b: Overview of the principles of the OMD

**Principles of the newly developed objective nine gaze-direction measuring device (hereinafter called "OMD").** The external view of the OMD is shown in Figure 1a. The device can objectively measure the nine gaze-directions simultaneously in both eyes using gaze-detection technology. The device contains a partition, providing a dichoptic separation structure that can display separate images for the left and right eyes. The display is connected to an externally controlled computer. Gaze-detection adopts the pupil–corneal reflex method (8). Invisible near-infrared light is irradiated and images are captured by the camera. The captured images are processed by a computer to detect the pupil and corneal reflections (the first Purkinje image). Using these positions, we can calculate a straight line through the camera principal point and the center of corneal curvature using the method of Nagamatsu et al (9). Especially, they determined the center of corneal curvature by determining the intersection of these two straight lines using two cameras, while OMD estimates a plausible center of corneal curvature position on a straight line by using the corneal curvature radius, as a fixed value. Furthermore, by taking the refractive index inside the cornea and the distance between the cornea curvature and the pupil centers, as fixed values, the position of the center of the pupil and its optical axis can be determined based on geometric positioning. See the appendix for detailed derivation methods.

To determine the fixation point, it is necessary to determine the visual axis passing through the target and the fovea. The visual axis cannot be determined directly due to the deviation between the optical axis and the visual axis (10). Therefore, calibration of one point is performed for the subject, and the amount of deviation between the optical axis and visual axis is found. While the subject gazes at a certain point, an optical axis vector is obtained. At this time, since the visual axis is considered to be in the direction of a certain point, the amount of deviation between the optical axis vector and the visual axis vector, that is, the relative direction of the visual axis vector to the optical axis vector can be obtained. By keeping this, the visual axis vector can be estimated from the acquired optical axis vector.

The visual axis is calculated from the optical axis, and the amount of deviation and the point of intersection with the screen (projection plane) is used to determine the gaze point (Figure 1b). The device has a compact design; it does not require long testing distances, and the test can be conducted in a bright room. When measuring the right eye, only a black dot can be seen on the display for the left eye (visual angle 0.7°), and the patient is instructed to fix the vision on this fixation target (background is white). The position of the right eye is measured with the left eye remaining fixed at the position of the fixation target for a certain period of time. The sampling rate is 30 Hz. The actual measurement screen is shown on video file (appendix).

**Examination 1: Examination of** **correlation, addition error, and proportional error of the Hess screen test and the OMD.** The subjects of the study were 20 healthy young people (mean age 20.9 ± 0.8 years) with exodeviation demonstrated with a subjective Hess screen test (test distance: 1.4 m, dichoptic image separation: red–green separation), and without any ophthalmological diseases other than refractive error. Each of the nine gaze-directions were measured using a Hess screen test and with the OMD, and correlation, addition error, and proportional error were examined. The right eye was used for measurement. The measurement order was randomized.

Spearman’s rank correlation coefficient was used for statistical analysis of correlation, and Bland–Altman analysis was used for statistical analysis of addition error and proportional error (11). A significance level of less than 5% was considered to reflect a significant difference.

**Examination 2: Examination of reproducibility for the OMD.** Two consecutive measurements of the nine gaze-directions were taken with the OMD in 20 healthy young people (with subjects different from those of Examination 1) who demonstrated exodeviation with the OMD, to examine reproducibility. The right eye was used for measurement. The intraclass correlation coefficient (1,1) was used for statistical analysis of reproducibility, and Bland–Altman analysis was used for statistical analysis of addition error and proportional error (11). Spearman’s rank correlation coefficient was used for statistical analysis of correlation. A significance level of less than 5% was considered to reflect a significant difference.

**Examination 3: Measurements in patients with strabismus**. Each of the nine gaze-directions were measured using the Hess screen test and OMD for three patients diagnosed with strabismus based on a Cover Test for distance vision, and the results were compared. The measurement time for each patient (from calibration through to completion of measurements) was recorded for the OMD. The measurement order was randomized.

## Results

**Figure 2.**
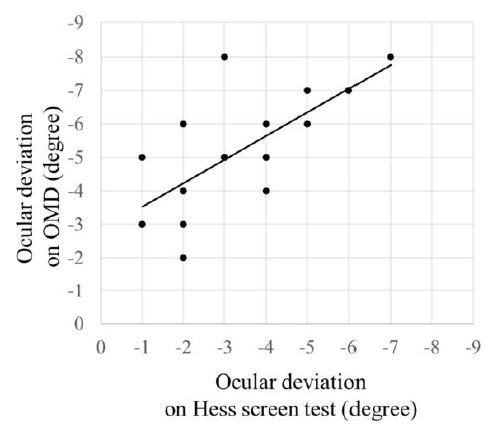
Comparison of the nine gaze-directions obtained using the Hess screen test and with the OMD.

**Examination 1: Examination of correlation, addition error, and proportional error for the Hess screen test and the OMD.** The nine gaze-directions in the Hess screen test and OMD are given in Table 1. The correlation between the Hess screen test and the OMD in the primary position is shown in Figure 2. Significant correlation was found for all nine gaze-directions between the Hess screen test and the OMD (center: P < 0.001, R^2^ = 0.50; top: P = 0.0020, R^2^ = 0.44; top right: P < 0.001, R^2^ = 0.71; right: P = 0.003, R^2^ = 0.47; bottom right: P < 0.001, R^2^ = 0.60; bottom: P < 0.001, R^2^ = 0.53; bottom left: P = 0.002, R^2^ = 0.54; left: P = 0.0073, R^2^ = 0.44; top left: P < 0.001, R^2^ = 0.64).

**Table 1.** Comparison of the nine gaze-direction measurements with the Hess screen test and the new objective OMD. The Hess screen test cannot be used for evaluating patients with suppression because the grid is displayed in one eye and the target is displayed in the other eye under red–green dichoptic separation (2). gaze-direction measuring device

Hess screen test		
-3.1±1.6°	-2.7±1.3°	-2.1±0.9°
-2.9±1.3°	-3.3±1.7°	-2.7±1.6°
-3.0±1.7°	-2.4±1.3°	-2.2±1.3°
OMD		
-4.7±2.0°	-5.2±1.7°	-5.0±1.3°
-4.9±1.3°	-5.1±1.7°	-5.1±1.8°
-5.0±1.9°	-4.6±1.5°	-4.5±2.0°

**Figure 3.**
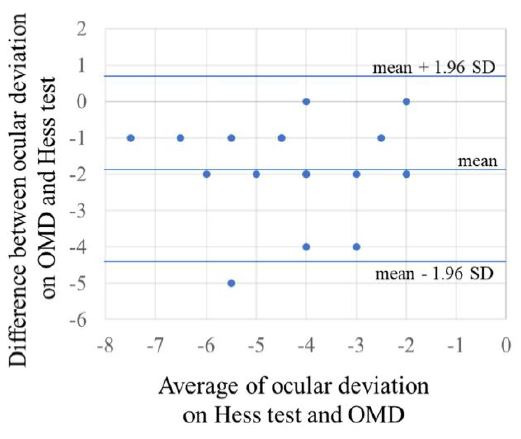
Bland–Altman analysis of the primary position in the Hess screen test and the OMD

The Bland–Altman analysis of the primary position in the Hess screen test and the OMD is shown in Figure 3. The OMD found significant addition error (exodeviation) compared to the Hess test (95% confidence interval center: -1.3 to -2.4, top: -1.9 to 3.0, top right: -2.6 to -3.2, right: -1.8 to -3.0, bottom right: -1.9 to -2.8, bottom: -1.7 to -2.7, bottom left: -1.3 to -2.6, left: -1.4 to -2.5, top left: -1.1 to -2.2). There was no significant proportional error in the Hess screen test and in the OMD (center: P = 0.69, top: P = 0.13, top right: P = 0.14, right: P = 0.30, bottom right: P = 0.84, bottom: P = 0.33, bottom left: P = 0.76, left: P = 0.98, top left: P = 0.11).

**Figure 4.**
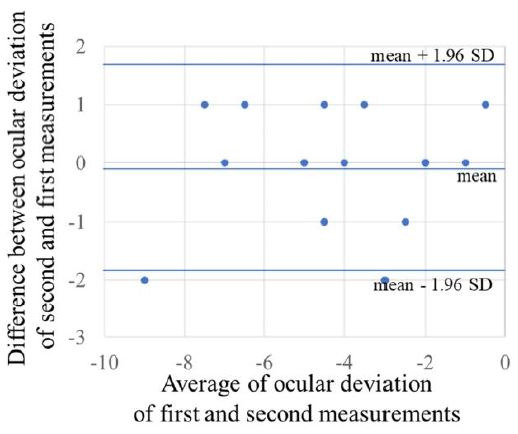
Bland–Altman analysis of the reproducibility of the OMD

**Examination 2: Examination of reproducibility for the OMD.** The first and second nine gaze-directions measurements obtained with the OMD are given in Table 2. The intraclass correlation coefficient (1,1) for the first and second measurements were center: 0.93, top: 0.90, top right: 0.86, right: 0.95, bottom right: 0.89, bottom: 0.90, bottom left: 0.85, left: 0.83, top left: 0.93, indicating good reproducibility. The Bland–Altman analysis of the first and second measurements are shown in Figure 4. There was no fixed error in the first and second measurements of the nine gaze-directions (95% confidence interval center: -0.5 to 0.3, top: -0.5 to 0.5, top right: -0.3 to 0.7, right: -0.2 to 0.5, bottom right: -0.6 to 0.5, bottom: -0.5 to 0.3, bottom left: -0.3 to 1.2, left: -0.4 to 0.7, top left: -0.7 to 0.1), and there was no proportional error (center: P = 0.83, top: P = 0.87, top right: P = 0.92, right: P = 0.85, bottom right: P = 0.15, bottom: P = 0.10, bottom left: P = 0.57, left: P = 0.99, top left: P = 0.77).

**Table 2.** Reproducibility of measurements obtained with the OMD

First Time		
-3.9±2.5°	-3.8±2.2°	-3.1±2.1°
-3.9±2.1°	-3.9±2.3°	-3.6±2.6°
-4.0±2.7°	-3.4±2.2°	-3.3±2.2°
Second Time		
-4.2±2.4°	-3.8±2.1°	-2.9±2.1°
-3.7±2.1°	-4.0±2.4°	-3.4±2.6°
-3.5±2.9°	-3.5±1.8°	-3.3±2.5°

**Figure 5.**
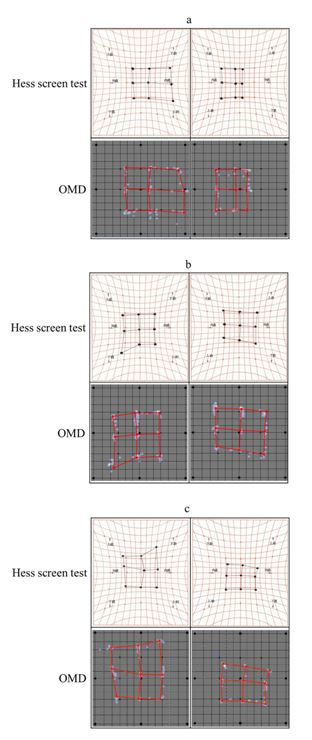
a: Measurements obtained using the Hess screen test and OMD for right eye abducens nerve paralysis; b: Measurements obtained using the Hess screen test and OMD for right eye trochlear nerve paralysis; c: Measurements obtained using the Hess screen test and OMD for right eye sursumvergence disorder, associated with thyroid-associated ophthalmopathy

**Examination 3: Measurements in patients with strabismus.** In the patient with right eye abducens nerve paralysis (Figure 5a), limited eye movement of the right eye and excessive movement of the left eye when looking to the right, which is associated with right eye abducens nerve paralysis, could be observed with both methods. Esodeviation was found in the primary position. The measurement time was 62 seconds.

In the patient with right eye trochlear nerve paralysis (Figure 5b), limited eye movement of the right eye and the excessive movement of the left eye when looking left and down, which is associated with right eye trochlear nerve paralysis, could be observed with both methods. R/L (upward deviation of right eye) was also observed for the primary position. The measurement time was 68 seconds.

In the patient with right eye sursumvergence disorder (Figure 5c), the limited eye movement of the right eye and the delayed excessive movement of the left eye when looking up, which is associated with thyroid-associated ophthalmopathy, could be observed with both methods. R/L was also observed for the primary position. The measurement time was 60 seconds.

The delayed excessive movement could be measured in all three patients using the OMD, similar to the Hess screen test. The results of the OMD deviated laterally as compared to the Hess screen test.

## Discussion

In this study, we compared the measurements of eye movement deviation in patients with strabismus, obtained using the Hess test and with a newly developed eye-tracking device, for each of the nine gaze-directions. With this device, we found that it was possible to measure the nine-gaze-direction deviations objectively, and found a strong correlation with conventional subjective measurements methods.

**Figure 6.**
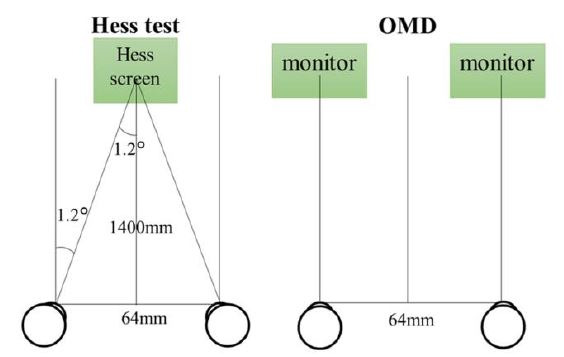
Difference in eye position between Hess test and OMD without deviation

OMD showed a horizontal addition error compared to Hess screen test. The reason for this may be the difference in dichoptic viewing methods between the Hess test and OMD. As shown in Figure 6, since the test distance of the Hess test is 1.4 m, assuming that the interpupillary distance is 64 mm, the 1.2-degree convergence position is evaluated as no deviation. On the other hand, in OMD, since the monitors are located in front of both eyes, the state of far distance view without convergence is evaluated as 0°. Furthermore, the conventional method performs dichoptic viewing with red–green glasses, and the grid can be seen with the fixing eye, while the green target can be seen with the tested eye. Therefore, the patient’s own attempt to overlap the targets into a single point causes a slight fusional movement, despite the dichoptic viewing. Therefore, the patient’s own attempt to overlap the targets into a single point causes a slight fusional movement, despite the dichoptic viewing. Conversely, measurements with the OMD only require display of the target in one eye (the fixing eye). Therefore, there is absolutely no fusional movement, so that latent ocular deviation may be more clearly detected as compared to the conventional method conditions. For these reasons, it is considered that horizontal addition error was detected in OMD as compared to Hess test.

However, the relative value, not the absolute value, is important for identifying ocular motility paralysis, and thus, this fixed deviation would not affect interpretation of the results as compared to the conventional method.

The measurements obtained with the device had good reproducibility. The OMD could enable measurements under strong dichoptic viewing and at a high sampling rate, which rendered it stable and enabled the measurement of ocular deviation.

Moreover, measurements in patients with strabismus demonstrated excessive movement of the healthy eye relative to limited movement of the affected eye (12), similar to that observed with the conventional method. There was a slight lateral deviation with the device as compared with the conventional method, but this is thought to be due to the effect of the aforementioned differences in the dichoptic viewing methods. The measurement time with the device was also extremely low, with measurements completed in about 1 minute for all subjects. Shortening the examination time can reduce the burden on the patient and also makes this approach amenable to screening scenarios.

The OMD can further be used for measurement of patients with suppression. In Hess screen tests to date, the grid is displayed in one eye and the target is displayed in the other eye, which makes measurements impossible in patients with suppression. However, with the OMD, the target is only displayed in one eye, so that the nine gaze-directions can be measured even in patients with suppression.

In conclusion, the newly developed device can objectively measure eye movements in the nine gaze-directions with a high degree of reproducibility. The results so obtained are strongly correlated with those obtained using the conventional method and measurements can be obtained rapidly. We now plan to use this device for various applications, including electro-oculography and nystagmus tests.

### Ethics and Conflict of Interest

The authors declare that the contents of the article are in agreement with the ethics described in http://biblio.unibe.ch/portale/elibrary/BOP/jemr/ethics.html and that there is no conflict of interest regarding the publication of this paper.

### Acknowledgements

This study was supported by a grant from Kitasato University School of Allied Health Sciences (Grant-in-Aid for Research Project, No. 2019-1040).
