# Supplementary material for: Objective measurement of nine gaze-directions using an eye-tracking device
Source: J Eye Mov Res. 2020 Oct 6;13(6):10.16910/jemr.13.6.4. doi: 10.16910/jemr.13.6.4 (PMC8015013; doi:10.16910/jemr.13.6.4)
Supplement: Supplementary file 1 [file jemr-13-06-d-SD1-01.pdf]

This appendix describes how OMD derives the corneal center of curvature and the optical axis.

1. An invisible near-infrared light is irradiated into the eye and the camera captures the image.

2. The images are processed by computer to detect the pupil and cornea reflection.

3. Using the method of Nagamatsu et al. (cited in Equation 2.2 (1) and Equation (2)) (Nagamatsu et al., 2008), we estimate a straight line passing through the main point of the camera and center of corneal curvature.

4. Assume that there is a center of corneal curvature at some position on the estimated straight line. Figure A shows the positional relationship at this time. C is the position of the main camera point, M is the line estimated by 1, r is the radius of corneal curvature, and A is the position of the hypothetical center of corneal curvature.

5: Since the radius of corneal curvature r is a fixed parameter in this method, the light source reflection position P_0 on the cornea can be obtained from the center of corneal curvature A, the position C of the camera principal point, and the position P_0' of the Purkinje image on the image sensor.

6: Determine the line M_0 that passes through P_0 and bisects the angle $\angle CP_0L_0$.

7: For the other light source, L_1, repeat 3 and 4 to determine the line M_1.

8: We determine the intersection of M_0 and M_1 (or the closest point from both of the two lines) and let this be $\hat{A}$.

9: If A coincides with the location of the center of corneal curvature, then $\hat{A}$ coincides with A because both M_0 and M_1 pass through A. The position of the center of corneal curvature can be obtained by determining A, where $\|A - \hat{A}\|^2$ is the minimum.

10: Next, we determine the position of the pupil center. In this method, since corneal curvature radius r, refractive index inside the cornea, and the distance d between the center of corneal curvature and center of the pupil are fixed parameters, the pupillary image refractive position on the cornea, B'' is obtained from corneal curvature center A, position of the camera principal point C, and the position of the pupil center on the image sensor, B', and pupil center position B inside the cornea is also obtained. The positional relationship at this time is shown in Figure B.

11: The optical axis is obtained as a line passing through A and B.

Since the three parameters are fixed in a series of steps, the estimated results are subject to

error due to individual differences. The error appears in the scale of the gaze point position on the Hess screen and is compensated for using the data at fixation.

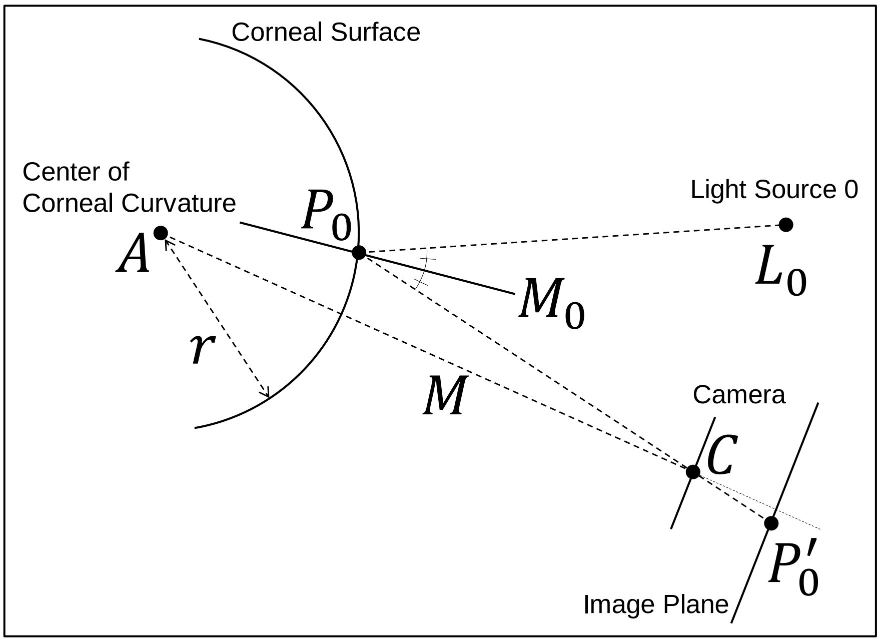

Figure A

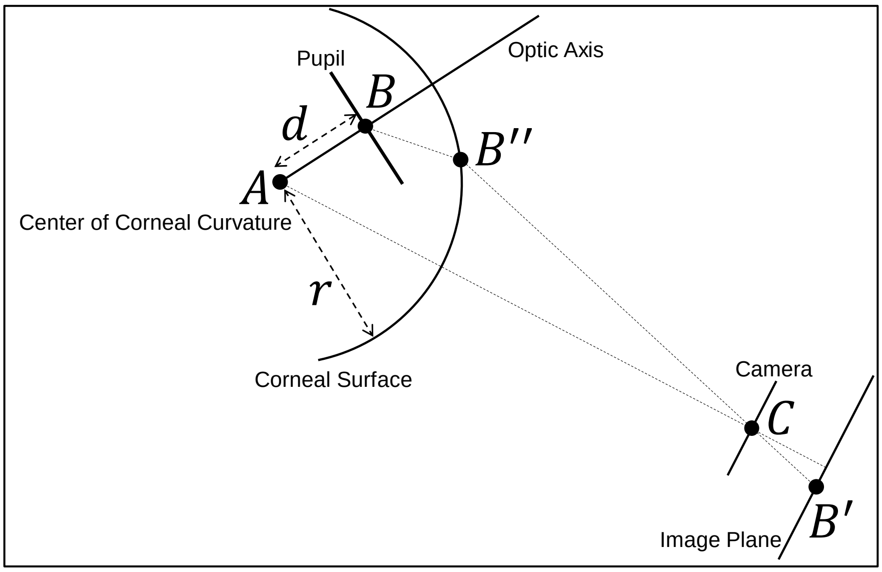

Figure B
